# Apical Hypertrophic Cardiomyopathy Mimicking Wellens Syndrome: A Diagnostic Challenge in a Patient Presenting With Recurrent Angina

**DOI:** 10.7759/cureus.113856

**Published:** 2026-08-03

**Authors:** Marco Antonio Rodríguez Sánchez, Jennifer Jocelyn Jacobo García, José Alberto Ayon Martinez, Itxel Nalleli Solorio Armenta, Yolanda Mercado Heredia, Alan Nemesio Barrios Angulo, Mario Castillo Fuentes

**Affiliations:** 1 Department of Cardiology, General Hospital of Culiacán, Autonomous University of Sinaloa, Culiacán, MEX

**Keywords:** apical hypertrophic cardiomyopathy, echocardiography, giant t-wave inversion, wellens syndrome, yamaguchi syndrome

## Abstract

Apical hypertrophic cardiomyopathy (ApHCM), also known as Yamaguchi syndrome, is an uncommon variant of hypertrophic cardiomyopathy that may mimic acute coronary syndromes because of its characteristic electrocardiographic abnormalities.

We report the case of a 66-year-old man with hypertension, type 2 diabetes mellitus, and active tobacco use who presented with recurrent exertional chest pain and giant symmetric T-wave inversion in leads V2-V6, producing a Wellens-like electrocardiographic pattern that mimicked Wellens syndrome and initially raised suspicion for critical proximal left anterior descending (LAD) artery disease. Coronary angiography demonstrated no significant obstructive coronary artery disease. Transthoracic echocardiography subsequently revealed localized apical hypertrophy with a maximal wall thickness of 14 mm and the characteristic spade-shaped left ventricular cavity, establishing the diagnosis of ApHCM. Treatment with bisoprolol, telmisartan, and atorvastatin resulted in complete symptom resolution, and the patient remained asymptomatic during follow-up.

This case highlights the importance of considering ApHCM in the differential diagnosis of patients presenting with giant T-wave inversion and suspected acute coronary syndrome, as recognition of its characteristic electrocardiographic and echocardiographic features can facilitate an accurate diagnosis and help avoid misinterpreting Wellens-like electrocardiographic patterns as true Wellens syndrome.

## Introduction

Apical hypertrophic cardiomyopathy (ApHCM), also known as Yamaguchi syndrome, is an uncommon phenotypic variant of hypertrophic cardiomyopathy characterized by localized hypertrophy predominantly involving the left ventricular apex. It was first described by Sakamoto et al. in 1976 and subsequently characterized by Yamaguchi et al. in 1979 [1,2]. In Japan, this subtype has been reported more frequently in Asian populations, particularly in Japanese cohorts, where it accounts for approximately 13%-25% of hypertrophic cardiomyopathy cases in selected series. Lower frequencies have been reported in Western cohorts; however, these estimates vary according to ethnicity, referral population, diagnostic criteria, and imaging modality [3-5].

The diagnosis of ApHCM is primarily based on cardiac imaging. Conventional diagnostic criteria include a maximal apical wall thickness ≥15 mm together with an apical-to-basal left ventricular wall thickness ratio >1.5. However, patients with an apical wall thickness of 13-15 mm may also fulfill the diagnostic criteria when characteristic morphologic features, such as the spade-shaped left ventricular cavity, are present and no alternative cause of left ventricular hypertrophy is identified [5].

The electrocardiographic features of ApHCM are frequently distinctive and include left ventricular hypertrophy voltage criteria and giant negative T-wave inversion (>10 mm), most commonly involving the anterior precordial leads. In selected cohorts, giant T-wave inversion has been reported in up to 93% of patients; however, its prevalence may vary across different populations and may not reflect the full contemporary phenotypic spectrum of ApHCM. Nevertheless, when present, it remains an important diagnostic clue [4,5]. In addition, imaging studies typically demonstrate the characteristic spade-shaped configuration of the left ventricular cavity ("ace-of-spades" appearance), a characteristic, but not mandatory, morphological feature of ApHCM that may support the diagnosis in the appropriate clinical context [2,6].

Despite these characteristic features, ApHCM may represent a significant diagnostic challenge because its clinical presentation and electrocardiographic abnormalities can mimic acute coronary syndromes, particularly Wellens syndrome, which is classically associated with critical proximal left anterior descending (LAD) artery stenosis. Deep, symmetric T-wave inversion in both conditions may lead to diagnostic confusion and potentially unnecessary invasive investigations if the diagnosis is not carefully considered [7,8]. Distinguishing these conditions is clinically important because their pathophysiology, therapeutic approach, and prognostic implications differ substantially [8,9].

We present the case of a patient with recurrent exertional angina and a Wellens-like electrocardiographic pattern suggestive of high-risk myocardial ischemia, who was found to have angiographically normal epicardial coronary arteries and characteristic echocardiographic evidence of ApHCM. This case highlights the diagnostic challenge posed by this uncommon presentation and underscores the importance of integrating clinical, electrocardiographic, coronary angiographic, and echocardiographic findings to establish the correct diagnosis. 

## Case presentation

A 66-year-old male farmer with a history of long-standing hypertension (15 years), type 2 diabetes mellitus (10 years), and active tobacco use (40 pack-years) presented with a two-week history of recurrent exertional chest pain. He denied dyspnea, palpitations, syncope, orthopnea, or lower-extremity edema. His functional status corresponded to New York Heart Association (NYHA) functional class I, and prior to symptom onset, he remained physically active in his daily work. The initial episodes consisted of oppressive retrosternal chest pain of moderate-to-severe intensity (7/10 on the visual analog scale), without radiation or associated adrenergic symptoms, which resolved spontaneously.

Over the following days, he developed recurrent exertional angina precipitated by moderate-to-high physical activity, with episodes lasting less than five minutes and resolving with rest. Due to the persistence and increasing frequency of symptoms, he sought cardiology evaluation.

**Figure 1:**
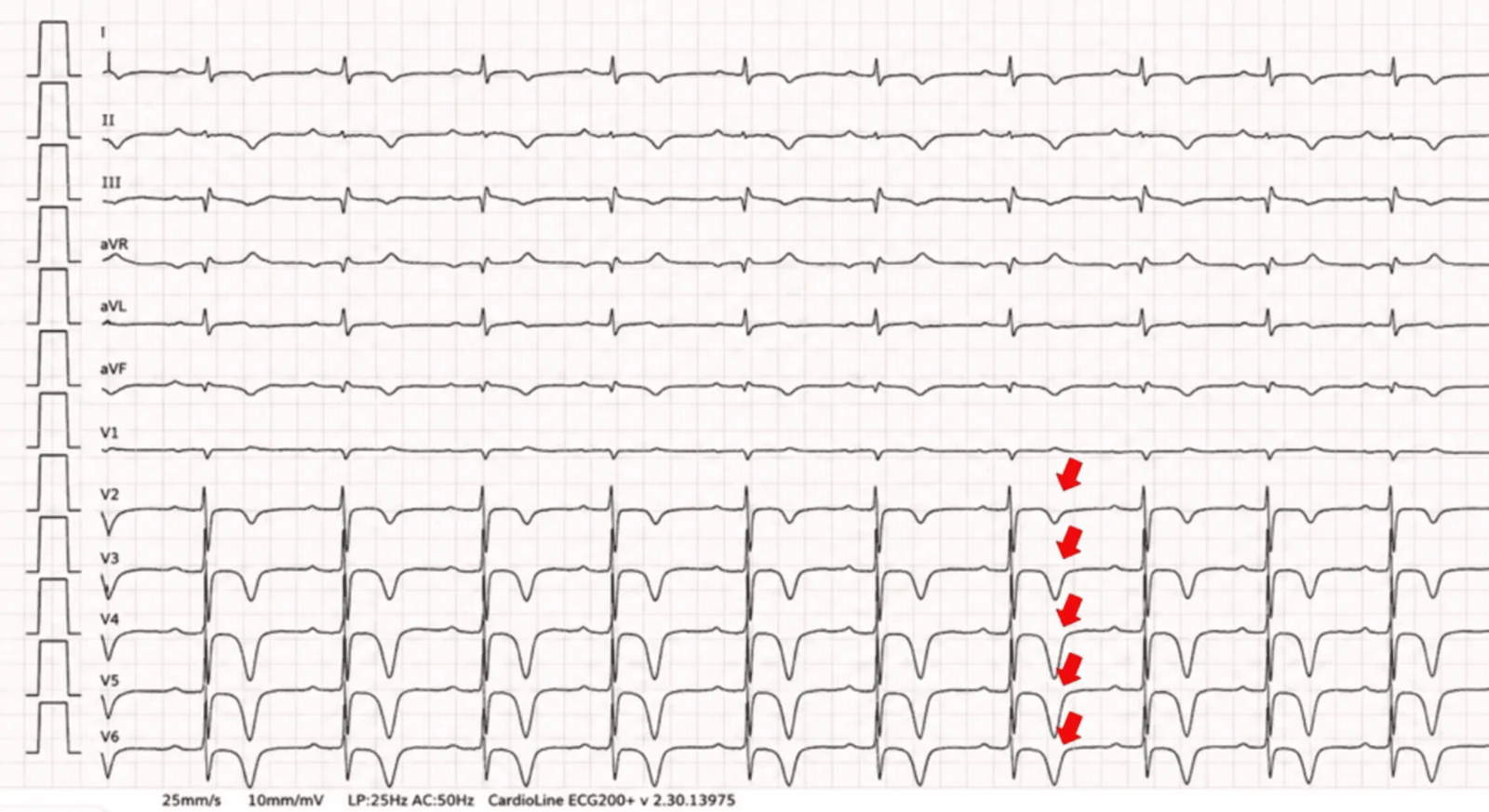
Admission electrocardiogram Twelve-lead electrocardiogram showing sinus rhythm with voltage criteria of left ventricular hypertrophy and marked symmetrical inversion of the giant T wave (>10 mm) affecting the anterior precordial leads (V2-V6), initially interpreted as an electrocardiographic pattern similar to Wellens' (red arrows).

Initial electrocardiographic assessment revealed sinus rhythm with voltage criteria for left ventricular hypertrophy and marked symmetric T-wave inversion involving the anterior precordial leads. T-wave depth exceeded 10 mm in leads V2-V6, consistent with giant T-wave inversion, initially interpreted as a Wellens type B pattern (Figure 1), raising suspicion for critical proximal LAD artery disease and high-risk unstable angina. Accordingly, the patient was referred to our institution for further evaluation and management.

On admission, laboratory studies revealed low-density lipoprotein (LDL) cholesterol 79 mg/dL, total cholesterol 127 mg/dL, triglycerides 86 mg/dL, glucose 101 mg/dL, urea 52 mg/dL, creatinine 0.97 mg/dL, sodium 137 mmol/L, potassium 4.6 mmol/L, hemoglobin 12 g/dL, platelet count 251 × 10³/µL, leukocyte count 6.4 × 10³/µL, high-sensitivity troponin <0.03 ng/mL, and B-type natriuretic peptide (BNP) 18 pg/mL (Table 1). 

**Table 1:** Initial laboratory findings on admission Initial laboratory findings on admission. Reference ranges may vary according to laboratory methods and assay-specific standards. Troponin cut-off values may differ according to the assay used at each institution*.

Laboratory parameter	Result	Reference range
Hemoglobin	12 g/dL	13.5-17.5 g/dL
Leukocyte count	6.4 × 10³/µL	4.0-10.0 × 10³/µL
Platelet count	251 × 10³/µL	150-450 × 10³/µL
Glucose	101 mg/dL	70-100 mg/dL
Urea	52 mg/dL	15-45 mg/dL
Creatinine	0.97 mg/dL	0.6-1.2 mg/dL
Sodium	137 mmol/L	135-145 mmol/L
Potassium	4.6 mmol/L	3.5-5.0 mmol/L
Total cholesterol	127 mg/dL	<200 mg/dL
Low-density lipoprotein (LDL) cholesterol	79 mg/dL	<100 mg/dL
Triglycerides	86 mg/dL	<150 mg/dL
High-sensitivity cardiac troponin	<0.03 ng/mL	<0.03 ng/mL*
B-type natriuretic peptide (BNP)	18 pg/mL	<100 pg/mL

**Figure 2:**
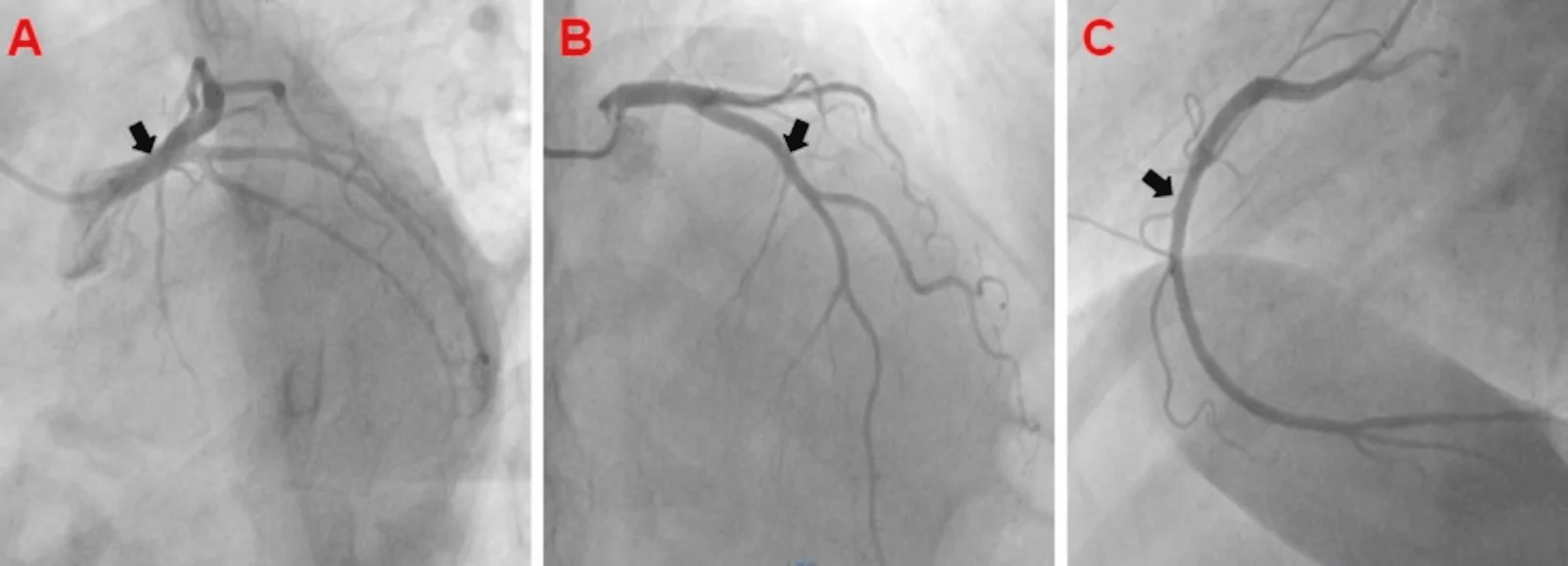
Coronary angiography Diagnostic coronary angiography demonstrating angiographically normal epicardial coronary arteries with no evidence of obstructive coronary artery disease, excluding the presence of critical proximal left anterior descending artery stenosis despite the initial electrocardiographic findings suggestive of a Wellens-like pattern. (A) Left main coronary artery. (B) Left anterior descending and left circumflex arteries. (C) Right coronary artery. Black arrows indicate the major epicardial coronary arteries, all exhibiting preserved luminal caliber without angiographically significant stenosis.

Given the high-risk clinical presentation and electrocardiographic findings suggestive of myocardial ischemia, the patient underwent invasive coronary angiography. The study demonstrated no significant angiographic coronary artery disease, excluding obstructive coronary disease as the underlying etiology of the patient’s symptoms and electrocardiographic abnormalities (Figure 2).

**Figure 3:**
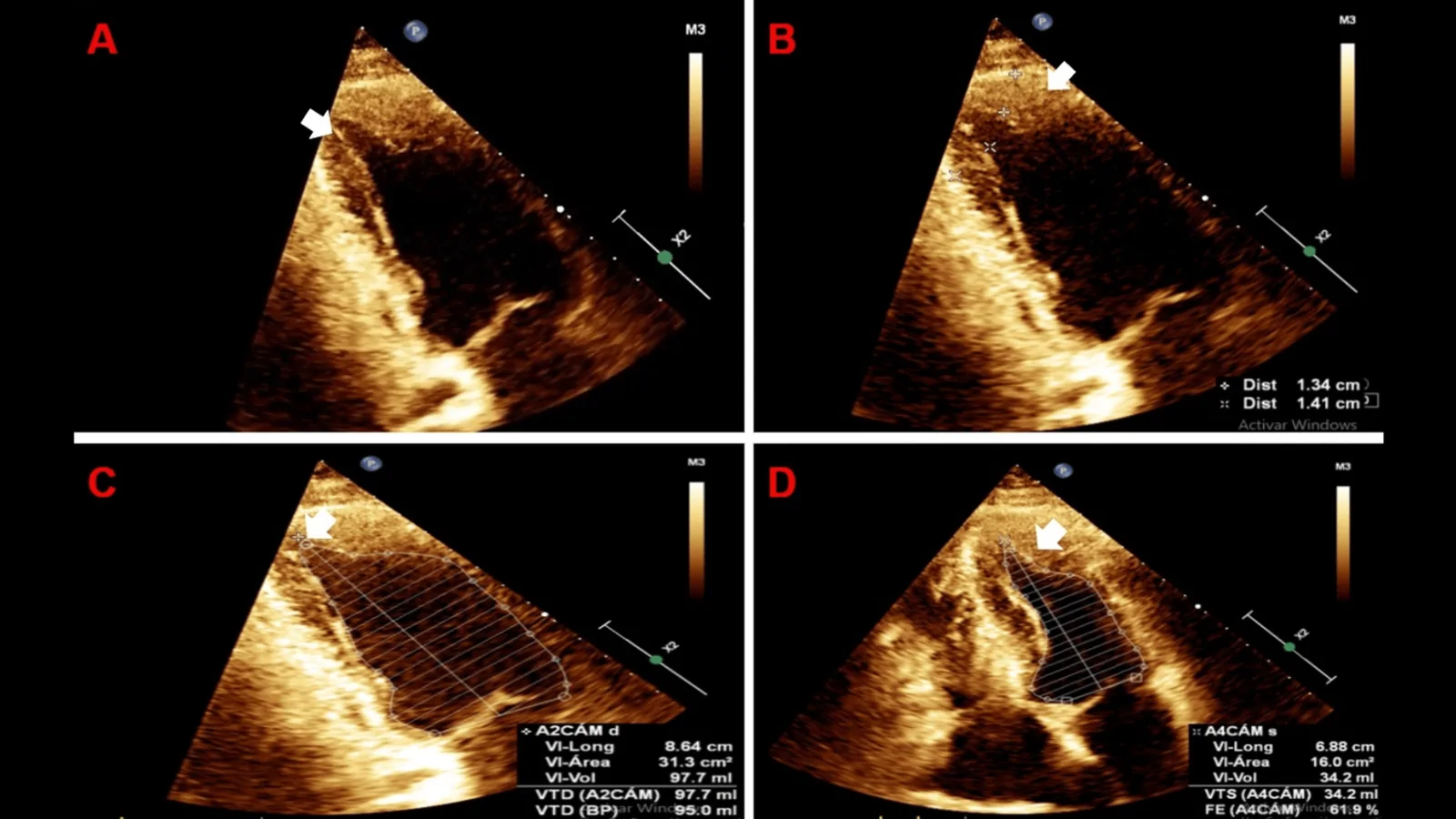
Transthoracic echocardiography Transthoracic echocardiography demonstrating focal hypertrophy confined to the left ventricular apex (A, white arrow), with a maximal apical wall thickness of 14 mm (B, white arrow). Apical four-chamber views reveal the characteristic spade-shaped left ventricular cavity ("ace-of-spades" appearance) during end-diastole (C and D, white arrows), a hallmark of apical hypertrophic cardiomyopathy (Yamaguchi syndrome).

Subsequent transthoracic echocardiography demonstrated localized left ventricular apical hypertrophy with a maximal apical wall thickness of 14 mm and an apical-to-basal wall thickness ratio of 1.75. Interventricular septal and posterior wall thicknesses measured 9 mm each. Left atrial volume index was 30 mL/m². The characteristic spade-shaped left ventricular cavity ("ace-of-spades" appearance) was observed in the apical four-chamber view. No resting left ventricular outflow tract obstruction was identified. Left ventricular systolic function was preserved, with an estimated ejection fraction of 55%. Doppler assessment demonstrated grade I diastolic dysfunction with normal filling pressures (average E/e′ = 10). No significant valvular abnormalities were identified (Figure 3).

Considering the characteristic echocardiographic findings, the presence of giant T-wave inversion on electrocardiography, and the absence of obstructive coronary artery disease, a diagnosis of ApHCM (Yamaguchi syndrome) was established.

Medical therapy was initiated with bisoprolol 5 mg once daily, telmisartan 80 mg once daily, and atorvastatin 40 mg once daily, in addition to continuation of baseline treatment for chronic comorbid conditions. The patient remained hospitalized for five days, during which he experienced complete resolution of anginal symptoms without complications. He was subsequently discharged with outpatient cardiology follow-up.

Following hospital discharge, the patient was followed in the outpatient cardiology clinic every 30 days. During six months of follow-up, he remained asymptomatic, with no recurrence of chest pain or exertional symptoms, good tolerance to medical therapy, and no evidence of heart failure, clinically significant arrhythmias, or other cardiovascular complications.

## Discussion

ApHCM is a distinctive morphologic subtype of hypertrophic cardiomyopathy characterized by predominant involvement of the left ventricular apex. Initially described by Sakamoto et al. and later further characterized by Yamaguchi et al., this entity has been reported more frequently in Asian populations, accounting for approximately 13%-25% of hypertrophic cardiomyopathy cases in Japan, whereas its prevalence in Western populations remains substantially lower [1-3]. Despite historically being considered a relatively benign condition, increasing evidence suggests that ApHCM may be associated with adverse cardiovascular outcomes, including arrhythmias, heart failure, thromboembolic events, and sudden cardiac death (SCD) [4,8,9].

Electrocardiographic abnormalities remain among the most characteristic findings of ApHCM. Giant negative T-wave inversion, generally defined as T-wave inversion exceeding 10 mm in precordial leads, has been reported in up to 93% of patients and frequently coexists with electrocardiographic criteria suggestive of left ventricular hypertrophy [2,4,5]. In the present case, the patient demonstrated marked symmetric giant T-wave inversion involving leads V2-V6, initially interpreted as a Wellens-like pattern and raising concern for critical proximal LAD artery disease.

The differentiation between ApHCM and Wellens syndrome represents an important clinical challenge because both conditions may present with deep symmetric T-wave inversion in the anterior precordial leads. However, Wellens syndrome classically reflects critical proximal LAD stenosis and represents a pre-infarction state associated with a high risk of extensive anterior myocardial infarction if left untreated. In contrast, giant T-wave inversion in ApHCM reflects abnormal ventricular repolarization secondary to localized apical myocardial hypertrophy rather than acute coronary ischemia [7,8]. Failure to recognize this distinction may lead to diagnostic uncertainty, unnecessary invasive procedures, and delays in establishing the correct diagnosis.

In our patient, the combination of recurrent anginal symptoms and a Wellens-like electrocardiographic pattern initially supported a working diagnosis of high-risk unstable angina. However, invasive coronary angiography demonstrated the absence of significant obstructive coronary artery disease, excluding critical proximal LAD involvement. Subsequent transthoracic echocardiography revealed localized apical hypertrophy with the characteristic spade-shaped configuration of the left ventricular cavity ("ace-of-spades" appearance), a hallmark imaging feature of ApHCM [2,4,6]. Collectively, these findings established the diagnosis of ApHCM (Yamaguchi syndrome).

Although the maximal apical wall thickness in our patient was 14 mm, which is below the conventional threshold of 15 mm for hypertrophic cardiomyopathy, the diagnosis of ApHCM was supported by an apical-to-basal wall thickness ratio of 1.75, the characteristic spade-shaped left ventricular cavity, giant T-wave inversion, and the absence of an alternative cause of left ventricular hypertrophy, consistent with current diagnostic recommendations [5,9].

Cardiac magnetic resonance (CMR) imaging was not performed in this patient. Although the diagnosis of ApHCM was strongly supported by the characteristic echocardiographic findings, including an apical-to-basal wall thickness ratio of 1.75, the typical spade-shaped left ventricular cavity, giant T-wave inversion on electrocardiography, and the absence of obstructive coronary artery disease, CMR is recommended by current guidelines for the comprehensive evaluation of hypertrophic cardiomyopathy because it allows more accurate assessment of myocardial morphology, late gadolinium enhancement as a marker of myocardial fibrosis, and the detection of apical aneurysms. Therefore, the absence of CMR represents a limitation of this case [8,9].

Current guidelines emphasize the role of multimodality imaging in the diagnosis and risk stratification of hypertrophic cardiomyopathy. Although CMR imaging has become an important diagnostic tool due to its ability to detect myocardial fibrosis and define the extent of hypertrophy, echocardiography remains the initial imaging modality and often provides sufficient diagnostic information when characteristic features are present [8,9]. In the present case, despite the absence of CMR imaging, the diagnosis was supported by the characteristic electrocardiographic and echocardiographic findings, together with the exclusion of obstructive coronary artery disease.

Following the diagnosis of ApHCM, contemporary guidelines recommend comprehensive risk stratification for SCD, including assessment of clinical risk factors, ambulatory electrocardiographic monitoring to detect nonsustained ventricular tachycardia, and multimodality imaging when appropriate. Implantable cardioverter-defibrillator (ICD) therapy should be considered in patients with established high-risk features according to current guideline recommendations. In the present case, the patient had no history of unexplained syncope, documented ventricular arrhythmias, left ventricular systolic dysfunction, or other recognized high-risk features at the time of diagnosis; therefore, conservative management with medical therapy and clinical follow-up was considered appropriate [8,9].

Despite the absence of obstructive epicardial coronary artery disease, patients with ApHCM may develop exertional angina as a consequence of coronary microvascular dysfunction. Hypertrophied myocardial fibers increase myocardial oxygen demand while simultaneously reducing subendocardial perfusion because of elevated intramyocardial pressure, impaired coronary flow reserve, and reduced capillary density. In addition, diastolic dysfunction and increased left ventricular filling pressures further compromise myocardial perfusion, particularly during exercise, resulting in a mismatch between oxygen supply and demand. These mechanisms provide a plausible explanation for the recurrent exertional angina observed in our patient despite angiographically normal coronary arteries [4,8,9].

Treatment strategies in ApHCM are generally directed toward symptom control and management of associated cardiovascular risk factors. Beta-blockers are frequently considered first-line therapy because they reduce myocardial oxygen demand and improve ventricular filling dynamics [8,9]. Following initiation of medical treatment with bisoprolol, our patient demonstrated complete symptom resolution and remained asymptomatic during outpatient follow-up.

This case highlights the importance of considering ApHCM in the differential diagnosis of patients presenting with deep T-wave inversion and suspected acute coronary syndrome. Recognition of this uncommon entity may facilitate timely diagnosis and prevent misinterpretation of electrocardiographic findings that may mimic high-risk ischemic conditions.

## Conclusions

ApHCM is an uncommon yet clinically important condition that may closely mimic high-risk ischemic syndromes because of its characteristic electrocardiographic manifestations. This case highlights the importance of recognizing ApHCM as an important differential diagnosis in patients presenting with recurrent exertional angina and Wellens-like electrocardiographic patterns despite angiographically normal epicardial coronary arteries. Increased awareness of this uncommon phenotype and the appropriate use of multimodality cardiac imaging may facilitate earlier diagnosis, avoid diagnostic misinterpretation and unnecessary interventions, and ensure appropriate risk stratification and long-term management according to current guideline recommendations.
